# Protective effects of intracerebroventricular adiponectin against olfactory impairments in an amyloid β_1–42_ rat model

**DOI:** 10.1186/s12868-021-00620-9

**Published:** 2021-03-02

**Authors:** Mara A. Guzmán-Ruiz, Amor Herrera-González, Adriana Jiménez, Alan Candelas-Juárez, Crystal Quiroga-Lozano, Claudia Castillo-Díaz, Erika Orta-Salazar, Diana Organista-Juárez, Sofía Díaz-Cintra, Rosalinda Guevara-Guzmán

**Affiliations:** 1 Departamento de Fisiología, Facultad de Medicina, Universidad Nacional Autónoma de México (UNAM), Mexico City, Mexico; 2 Departamento de Neurobiología del desarrollo y neurofisiología, Instituto de Neurobiología, Universidad Nacional Autónoma de México (UNAM), Querétaro, Mexico

**Keywords:** Adiponectin, Amyloid-beta, Alzheimer disease model, Hippocampus, Olfactory bulb, Olfactory dysfunction

## Abstract

**Background:**

Alzheimer’s disease (AD) is characterized by cognitive impairment that eventually develops into dementia. Amyloid-beta (Aβ) accumulation is a widely described hallmark in AD, and has been reported to cause olfactory dysfunction, a condition considered an early marker of the disease associated with injuries in the olfactory bulb (OB), the hippocampus (HIPP) and other odor-related cortexes. Adiponectin (APN) is an adipokine with neuroprotective effects. Studies have demonstrated that APN administration decreases Aβ neurotoxicity and Tau hyperphosphorylation in the HIPP, reducing cognitive impairment. However, there are no studies regarding the neuroprotective effects of APN in the olfactory dysfunction observed in the Aβ rat model. The aim of the present study is to determine whether the intracerebroventricular (i.c.v) administration of APN prevents the early olfactory dysfunction in an i.c.v Amyloid-beta_1–42_ (Aβ_1–42_) rat model. Hence, we evaluated olfactory function by using a battery of olfactory tests aimed to assess olfactory memory, discrimination and detection in the Aβ rat model treated with APN. In addition, we determined the number of cells expressing the neuronal nuclei (NeuN), as well as the number of microglial cells by using the ionized calcium-binding adapter molecule 1 (Iba-1) marker in the OB and, CA1, CA3, hilus and dentate gyrus (DG) in the HIPP. Finally, we determined Arginase-1 expression in both nuclei through Western blot.

**Results:**

We observed that the i.c.v injection of Aβ decreased olfactory function, which was prevented by the i.c.v administration of APN. In accordance with the olfactory impairment observed in i.c.v Aβ-treated rats, we observed a decrease in NeuN expressing cells in the glomerular layer of the OB, which was also prevented with the i.c.v APN. Furthermore, we observed an increase of Iba-1 cells in CA1, and DG in the HIPP of the Aβ rats, which was prevented by the APN treatment.

**Conclusion:**

The present study describes the olfactory impairment of Aβ treated rats and evidences the protective role that APN plays in the brain, by preventing the olfactory impairment induced by Aβ_1–42_. These results may lead to APN-based pharmacological therapies aimed to ameliorate AD neurotoxic effects.

**Supplementary Information:**

The online version contains supplementary material available at 10.1186/s12868-021-00620-9.

## Background

Alzheimer disease (AD) is a neurodegenerative condition, characterized by the accumulation of insoluble forms of amyloid-beta (Aβ) and intracellular aggregation of the phosphorylated microtubule-associated protein tau (p-TAU) [1, 2]. This results in neurodegeneration and cognitive impairment, including memory and olfactory dysfunction [3–6].

Olfactory impairment is considered an early marker for AD since odor sensibility, detection, and discrimination are significantly reduced in up to 70–95% of these patients [4–7]. Postmortem studies in humans and rodent models of AD show a direct correlation between the amount of soluble oligomeric forms of Aβ and the severity of olfactory impairments [7–10]. Accumulation of Aβ oligomers (AβO) in olfactory regions such as the olfactory epithelium, olfactory bulb (OB), anterior olfactory nucleus, piriform cortex, entorhinal cortex and hippocampus (HIPP) correlate with deficits in odor discrimination [6].

Conversely, disorders such as obesity, metabolic syndrome and type 2 diabetes are risk factors for the development of neurodegenerative pathologies such as AD [11, 12].

Adiponectin is an adipokine mainly secreted by mature white adipocytes, although it is also found in skeletal muscle, cardiac myocytes, and endothelial cells, and its secretion levels are inversely correlated with adiposity and insulin resistance [13].

There are several APN isoforms: (1) a full-length protein present in plasma that exists as a trimer, known as the low molecular weight isoform (LMW), (2) an hexamer, known as the middle molecular weight form (MMW) and (3) a 12- to 18-mer, constituting the high molecular weight (HMW) form. APN can also be found in a globular form, constituted by the C-terminal domain as a result from proteolytic cleavage. The LMW, the MMW and the globular forms of APN have been found in the central nervous system (CNS) [14–16].

APN is known for its pleiotropic actions, regulating a wide range of physiological processes such as: glucose and fatty acid metabolism, insulin sensitivity (in peripheral tissues and brain), the immune response and oxidative stress [13, 17, 18].

The effects of APN are carried out through the activation of adiponectin receptors 1 and 2 (AdipoR 1 and 2) [19–21] and the T-cadherin receptor, which is mainly expressed in the intima of the vascular endothelium [22, 23].

In the CNS, APN has an important neuroprotective role because it restores neuronal insulin signaling via the phosphorylation of the insulin receptor substrate-1 (IRS-1), enhancing cognitive performance in neurodegenerative models of AD [24, 25]. Furthermore, APN inhibits microglial pro-inflammatory responses to LPS and AβO, thus preventing cytotoxicity [26, 27]. In acute inflammatory events like cerebral ischemia, it inhibits the infiltration of immune cells into the brain parenchyma [28].

APN knockout (APN-KO) mice exhibit AD-like memory impairments and anxiety, accompanied by the accumulation of oligomers Aβ_1–42_ and p-TAU in the hippocampus [24]. A similar effect has been observed in the AdipoR1 knockdown (KD) [29], suggesting that impaired APN signaling in the brain might be involved in the pathogenesis of neurodegenerative diseases like AD.

In spite of the documented anti-inflammatory role of APN in the CNS, its role in the pathogenesis of neurodegenerative diseases is unclear. There is contrasting data regarding APN levels in different dementias, on one hand, APN is significantly higher in mild cognitive impairment and AD patients [30], on the other hand, the cerebrospinal fluid (CSF) levels of this adipokine are significantly low [31]. There is also conflicting data regarding increased CSF adiponectin in neurodegenerative diseases, which authors have suggested to be a counterregulatory effect to neurodegeneration [32]. Therefore, it is unclear whether this adipokine could exert a protective effect in neurodegenerative diseases such as AD.

In the present study, we hypothesized that the i.c.v. administration of APN would prevent the early olfactory dysfunction observed in a Aβ_1–42_ rat model, which would correlate with a decrease in Iba-1 expression in the HIPP and the prevention of the decrease of NeuN expressing cells in the glomerular layer in the OB in Aβ rats. For this, we first determined whether the intracerebroventricular (i.c.v.) administration of APN prevented the early olfactory dysfunction in an i.c.v. Amyloid-beta_1–42_ (Aβ_1–42_) rat model by using a battery of olfactory tests aimed to assess olfactory memory, discrimination and detection. In addition, we determined the number of cells expressing the neuronal nuclei (NeuN) marker, mainly located in the nuclei and perinuclear cytoplasm of mature neurons as an indication of neuronal differentiation. Next, we determined the number of microglial cells by using the ionized calcium-binding adapter molecule 1 (Iba-1) marker in the OB and HIPP We observed changes in this marker, suggesting a local inflammatory process in the brain. Finally, we determined the expression of the M2 microglial marker, Arginase 1 (ARG-1), observing an increase in the OB and the HIPP in response to APN i.c.v. administration.

## Results

### I.c.v. injection of adiponectin prevents olfactory memory impairment in the Aβ_1–42_ rat model

To determine olfactory memory in our Aβ rat model, we performed a social recognition test and habituation–dishabituation tests.

**Fig. 1:**
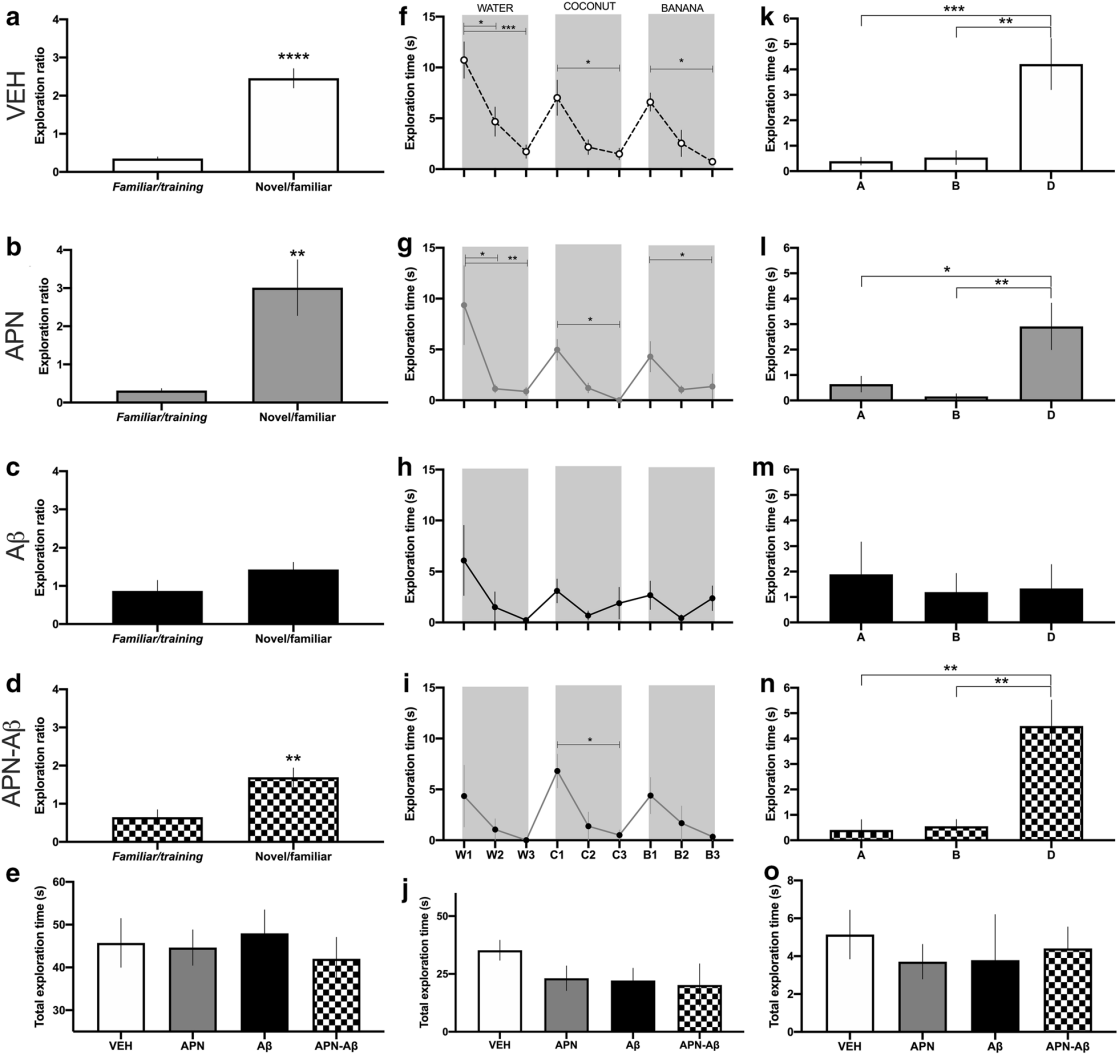
Olfactory memory and discrimination tests. Social recognition test: **a** exploration time in the social recognition test of vehicle group (VEH), **b** adiponectin and vehicle group (APN), **c** vehicle and Aβ (Aβ) and **d** adiponectin Aβ_1–42_ (APN–Aβ). The first bar denotes the exploration ratio between the juvenile 1 familiar/training and the second bar shows the exploration ratio between the novel/familiar, **e** total exploration time of both cages during the habituation trial (F_[8, 63]_ = 8.277, p < 0.0001). Habituation–dishabituation test: time smelling a cassette with different essences for **f** VEH (F_[8, 63]_ = 8.277, p < 0.0001), **g** APN (F_[8, 34]_ = 3.843, p < 0.0026), **h** Aβ (F_[8, 54]_ = 1.354, p < 0.2377, and **i** APN–Aβ (F_[8, 35]_ = 2.346, p < 0.0391), W1-3 corresponds to the three trials where the cassette was loaded with 50 µl of water; C1-3 with 50 µl of coconut extract; and B1-3 50 µl of banana extract, **j** total exploration time for all of the essences and water (F_[3, 19]_ = 1.455, p < 0.2584). Block test: time exploring each block for the groups **k** VEH (F_[2, 18]_ = 12.66, p < 0.0004), **l** APN (F_[2, 15]_ = 6.866, p < 0.0076), **m** Aβ (F_[2, 15]_ = 0.1347, p < 0.8750) and **n** APN–Aβ (F_[2, 21]_ = 12.68, p < 0.0002). First and second bars represent the time spent smelling two blocks that were placed in their own cage the night before. The third bar represents the time animals spent smelling a block from the cage of a different animal. **o** Total exploration time of the three blocks (F_[3, 23]_ = 0.1752 p < 0.9121). Data are presented as SEM and evaluated using a Student’s T test (**a**–**d**) or a One-way ANOVA with a post-hoc Tukey test (**e**–**o**). Asterisks represent significance (*p < 0.05, **p < 0.001)

In the social recognition test we measured the time that animals spent exploring a novel or a familiar juvenile rat and observed that VEH and APN treated rats spent significantly more time exploring a novel juvenile as compared to a familiar subject. In contrast, the Aβ injected animals spent the same time exploring both novel and familiar juveniles. Interestingly, rats injected with APN–Aβ, present similar exploration times as the VEH and APN groups (Fig. 1a–d).

To ensure that the differences in the time spent sniffing the familiar and unfamiliar juveniles were not due to motility impairments, we measured the exploring time of the void cages during the habituation trial, observing that the exploration time of all groups was similar (Fig. 1e).

To further evaluate olfactory memory, we employed the habituation–dishabituation test, which measures the time the animals spend smelling a scented histological cartridge in three consecutive trials for three different odors.

We observed that the Aβ group was unable to dishabituate in response to new odors as compared to the VEH and APN rats. In contrast, the animals administered with APN–Aβ only dishabituated with the coconut scent (Fig. 1f–i).

To determine if the differences in this test were not due to motility impairments, we compared the total amount of time that the animals sniffed each of the histological cartridges in all of the trials including water, observing no statistical differences (Fig. 1j). This demonstrates that all groups had the same motility and interest to explore the different cartridges. In addition, we observed that the habituation–dishabituation curves of intact and VEH were no different (Additional file 1: Fig. S1) demonstrating that the surgery did not interfere with the performance during this test.

### I.c.v. injection of adiponectin prevents odor discrimination impairment in an Aβ_1–42_ rat model

To evaluate odor discrimination, we performed the block test [23], which evaluates the capacity to recognize their own scent (blocks A–B) from the odor of another subject (block D).

We observed that the VEH and APN-treated groups explored block (D) significantly more as compared to their own (A–B). In contrast, the Aβ group spent the same time exploring all the blocks, while the APN–Aβ group investigated block D significantly more (Fig. 1k–n). These results suggest that APN prevents the odor discrimination impairments in the Aβ-treated rats. Importantly, total exploration time of the three blocks presented no significant differences amongst the groups, suggesting that the differences in block exploration were not attributed to motility impairments (Fig. 1o).

### Olfactory bulb immunohistochemical evaluation of NeuN and Iba-1

**Fig. 2:**
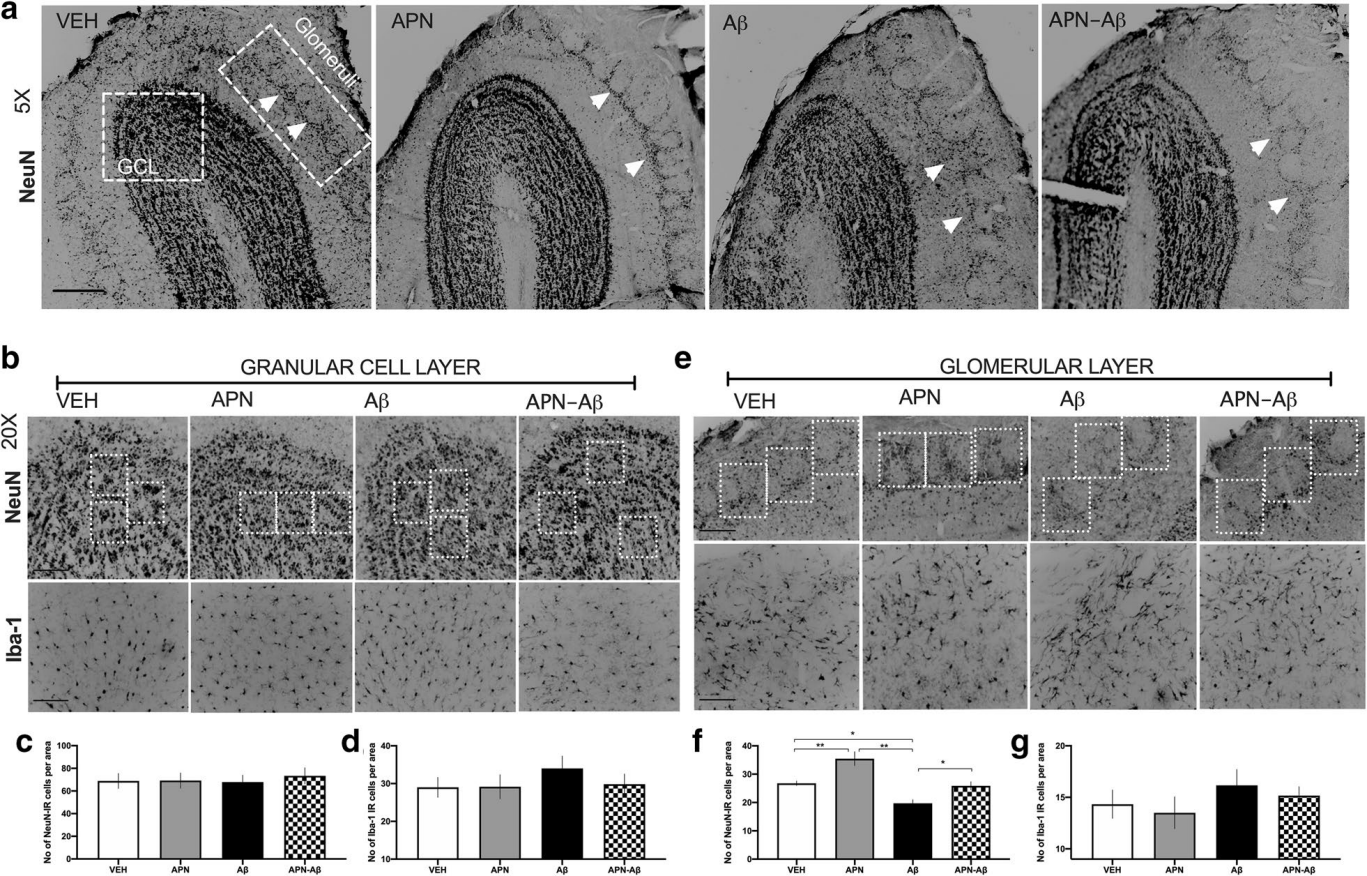
Immunohistological determination of NeuN and Iba-1 expression in the olfactory bulb. **a** Representative micrographies of the olfactory bulb showing the evaluated areas for NeuN and Iba-1, scale bar 250 µm; the arrows denote the region where glomeruli are disarranged. **b** Representative micrographies of NeuN (top) and Iba-1 (bottom) of the granular cell layer (GCL) with examples of the random squares (ROIs) quantified, scale bar 50 µm. **c** Number of NeuN expressing cells in the GCL for Vehicles (VEH), adiponectin (APN), Amyloid-β (Aβ and APN–Aβ injected rats (F_[3, 20]_  = 0.1, p < 0.94). **d** Number of Iba-1 positive cells per area of the GCL of the same groups (F_[3, 20]_ = 0.6336, p < 0.602). **e** Representative micrographies of the glomeruli with examples of the random squares (ROIs) quantified, scale bar 50 µm. **f** Number of NeuN expressing cells in the glomeruli per area of the same groups (F_[3, 68]_ = 15.93, p < 0.0001). **g** Circularity index of the glomeruli of the same groups (F_[3, 312]_ = 79.54, p < 0.0001). **h** Number of Iba-1 positive cells per area (F_[3, 20]_ = 0.7029, p < 0.5614. Data are presented as SEM and evaluated using a One-way ANOVA with a post-hoc Tukey test

To determine whether APN prevents NeuN and Iba-1 changes in primary regions involved in processing odors, we evaluated NeuN and Iba-1 expression in two different regions of the OB (Fig. 2a).

First, we evaluated the granular cell layer (GCL) and observed no changes in the number of cells expressing neither NeuN nor Iba-1 (Fig. 2b–d).

Next, we evaluated the number of NeuN expressing cells in the glomerular layer (GL) of the OB and detected an increase in the APN group, as well as a decrease in the Aβ treated rats. The decrease in NeuN-IR cells in the Aβ groups was prevented by APN (Fig. 2e, f). We did not observe changes in the amount of Iba-1 expressing cells in this region (Fig. 2g).

Finally, none of the areas beneath the glomerular layer including the external plexiform, the mitral/tufted cell area, the inner plexiform layer (data not shown), and the GL presented changes neither in the number of NeuN expressing cells nor in the thickness of the layers.

### Olfactory bulb Western blot evaluation of Aβ oligomers and Arginase-1

**Fig. 3:**
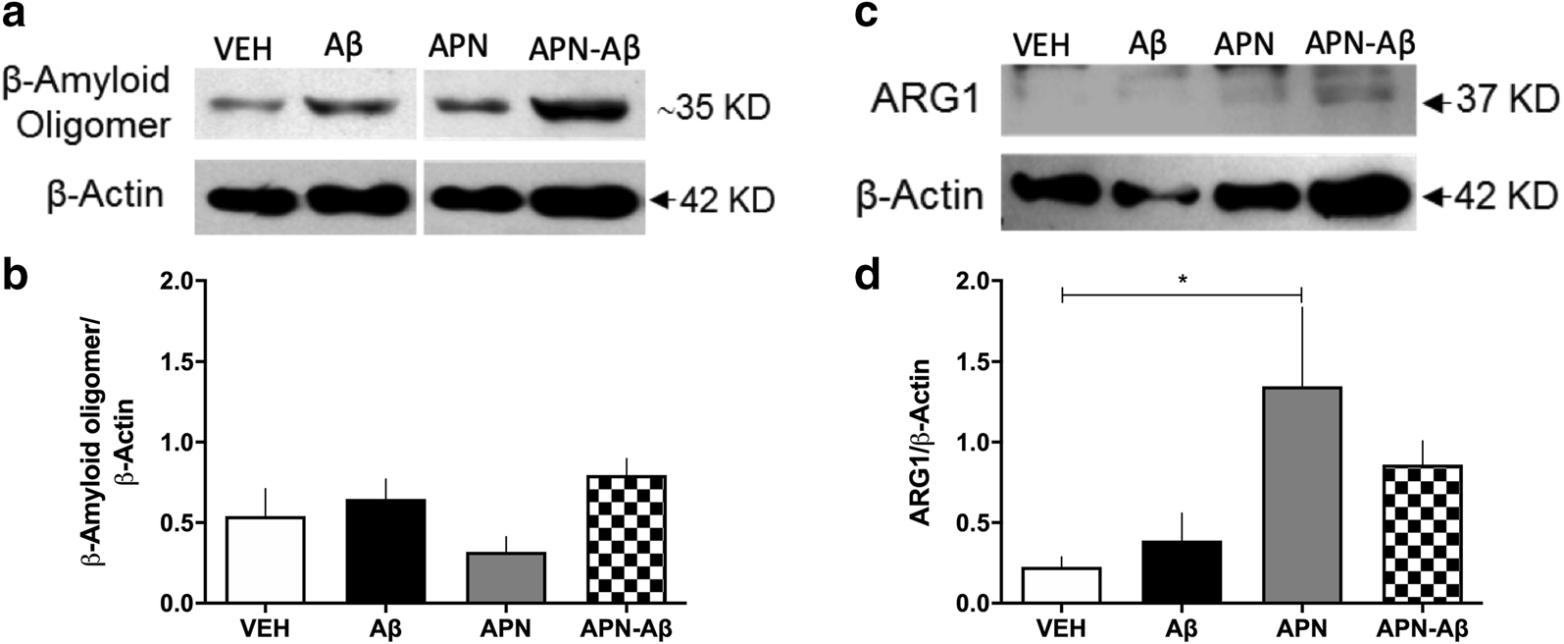
Protein expression in the olfactory bulb. **a** Representative Western blot of Amyloid β oligomer and β-Actin; **b** β-Amyloid/β-Actin ratio; **c** representative Western blot of Arginase1 (ARG1) and β-Actin; **d** ARG1/β-Actin ratio for the vehicles (VEH), adiponectin (APN), Amyloid β (Aβ) and APN–Aβ injected rats. Data are presented as SEM and evaluated using a One-way ANOVA with a post-hoc Tukey test

Since we observed a decrease in NeuN expression in the GL, we assessed if this effect could be related to the presence of AβO. We observed no differences in the amount of AβO in the OB of Aβ or APN–Aβ treated rats (Fig. 3a and b, Additional file 3: Fig. S3B, D).

Next, since APN is known for its anti-inflammatory effects [24], we evaluated Arginase-1 (ARG-1), a microglial marker mainly expressed when these cells polarize to a neuroprotective phenotype M2 [25]. We observed that rats injected only with APN significantly increased ARG-1 expression in the OB as compared to the VEH treated animals. In addition, the rats treated with APN–Aβ only presented a tendency to increase ARG-1 expression of this marker without reaching statistical significance (Fig. 3c and d, Additional file 4: Fig. S4A, B).

### Hippocampal immunohistochemical evaluation of NeuN and Iba-1

**Fig. 4:**
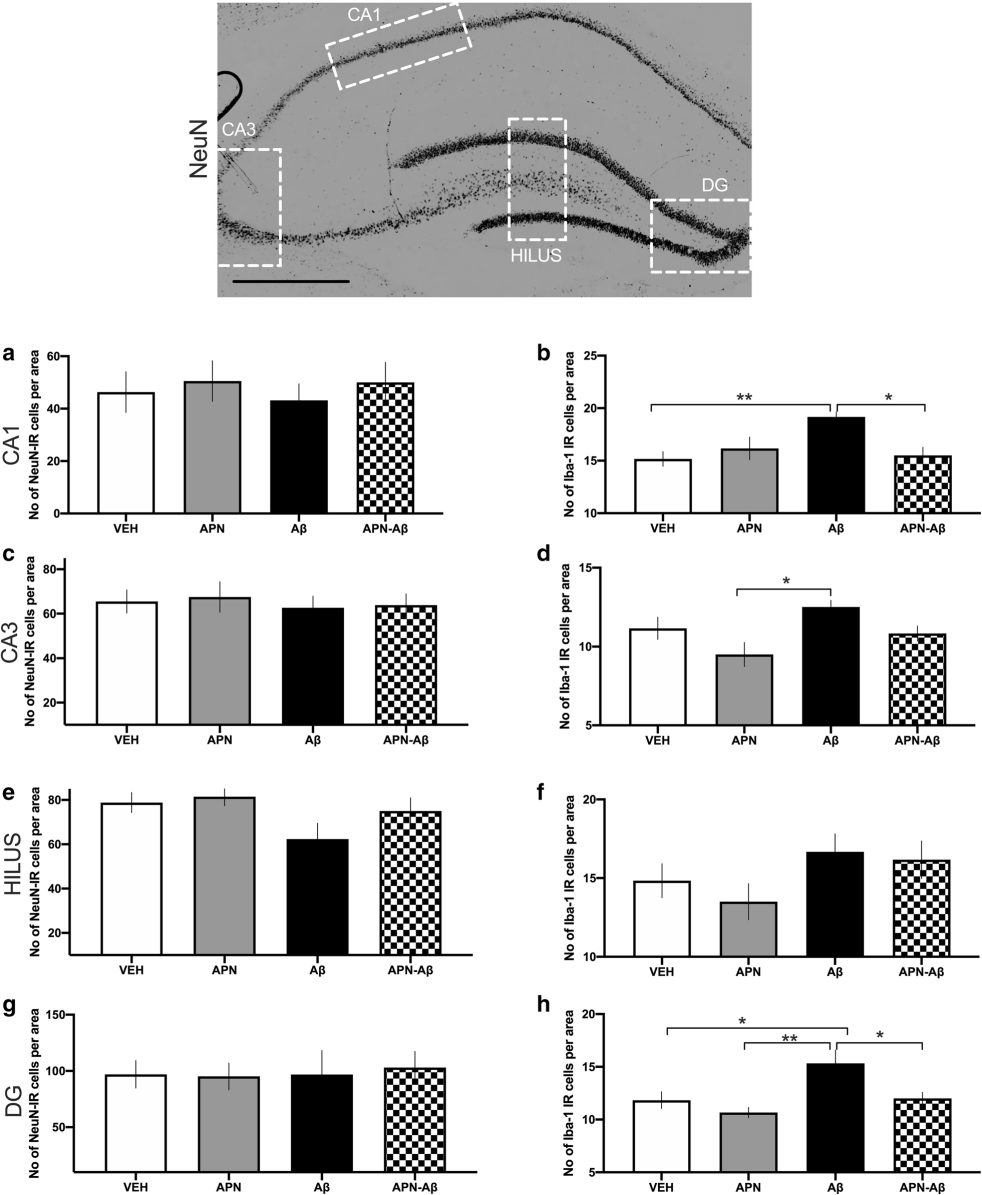
Hippocampal NeuN and Iba-1 expressing cells per area. In the top there is a representative micrography (×4) of the evaluated hippocampal regions (scale bar 200 µm) with the regions evaluated for NeuN and Iba-1. **a** CA1 NeuN (F_[3, 20]_ = 0.2144, p = 0.8852) and **b** Iba-1 (F_[3, 20]_ = 5.4, p = 0.0069) positive cells per area. **c** CA3 NeuN (F_[3, 20]_ = 0.1392, p = 0.9354) and **d** Iba-1 (F_[3, 20]_ = 4.08, p = 0.02) expressing cells per area. **e** Hippocampal hilus NeuN (F_[3, 20]_ = 0.2.338, p = 0.1042) and **f** Iba-1 (F_[3, 20]_ = 1.553, p = 0.2319) expressing cells per area. **g** Dentated Gyrus (DG) NeuN (F_[3, 20]_ = 0.05, p = 0.985) and **h** Iba-1 (F_[3, 20]_ = 5.785, p = 0.0051) expressing cells per area for the vehicles (VEH), adiponectin (APN), Amyloid-β (Aβ) and APN–Aβ injected rats. Data are presented as mean + SEM and evaluated using a One-way ANOVA with a post-hoc Tukey test. Asterisks represent significant differences (*p < 0.05, **p < 0.01). Higher magnifications of each region for both antibody stainings are presented in Additional file 1: Fig. S1

To determine if our APN treatment could prevent hippocampal damage caused by the administration of Aβ_,_ we performed a histological evaluation of the HIPP, where we determined the number of cells expressing NeuN and Iba-1. We did not find changes in the number of hippocampal NeuN expressing cells in any of the treatments (Fig. 4a, c, e, g).

Furthermore, we observed that Aβ administration induced a significant increase in Iba-1 positive cells in CA1, CA3 and dentate gyrus (DG). Only the CA1 and GD regions showed a significant reduction in the number of Iba1-positive cells as a result of the APN treatment prior to Aβ administration (Fig. 4b, d, h and Additional file 2: Fig. S2).

### Hippocampal Western blot evaluation of Aβ oligomers and Arginase-1

**Fig. 5:**
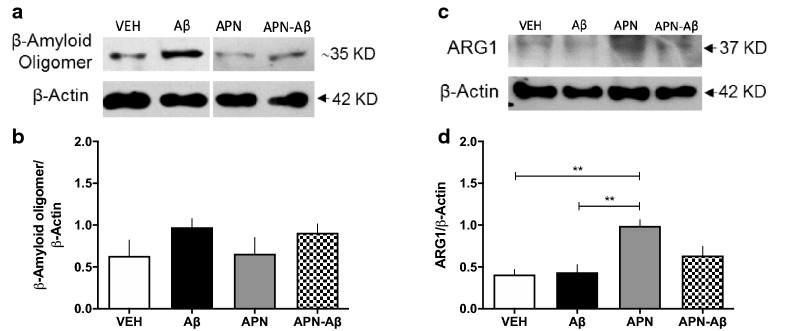
Protein expression in the hippocampus. **a** Representative western blot of β-Amyloid oligomer and β-Actin; **b** β-Amyloid/β-Actin ratio; **c** representative western blot of Arginase1 (ARG1) and β-Actin; **d** ARG1/β-Actin ratio for the vehicles (VEH), adiponectin (APN), Amyloid-β (Aβ) and APN–Aβ injected rats. Data are presented as SEM and evaluated using a One-way ANOVA with a post-hoc Tukey test

Since we observed changes in Iba-1 expression in the HIPP, we evaluated if this could be related to the presence of AβO. We observed no increase in AβO neither in the Aβ nor in the APN–Aβ treated animals (Fig. 5a and b, Additional file 3: Fig. S3A, C).

Finally, we evaluated the microglial marker ARG-1, and found a significant increase only in the APN group as compared to the VEH and Aβ treated animals.

Similar to the OB ARG-1 in the APN–Aβ rats there was a tendency to increase its expression without reaching significance (Fig. 5c and d, Additional file 4: Fig. S4A, B).

## Discussion

In the present work, we employed three behavioral tests to evaluate the olfactory performance of rats injected with Aβ_1–42_. We observed that rats administered with Aβ presented impairments in olfactory detection and memory, which were prevented with i.c.v. APN. These results are in agreement with previous studies reporting olfactory impairments in humans with different dementias and in rodent models of neurodegenerative diseases [6].

In the present study, we did not find significant changes in AβO accumulation in OB or HIPP. This is contrast with what has been reported in genetic models such as the AβPP/PS1 and Tg2576 where AβO accumulation appears at 3 months of age [6, 8, 10], or in chronic models, where AβO is accumulated in high quantities [33–36]. The lack of accumulation is probably due to brain clearance mechanisms, given that the infusions were performed in previously healthy animals [37, 38]. Although there are few reports showing the presence of Aβ_1–42_ 2–7 weeks after an acute i.c.v. administration, these measurements were performed by immunohistochemistry and not western blot like in the present study [39, 40]. Therefore, it is possible that the lack of visualization of the aggregates in this acute protocol are due to the visualization technique, since the dose of AβO was similar to ours.

We believe that, although the clearance rate is probably enough to prevent accumulation of AβO, it is unable to prevent AβO’s acute effects in the local circuitries of the OB that induce olfactory impairments.

The interconnections in the OB are complex, the glomerular layer receives excitatory inputs from olfactory sensory neurons that mainly contact periglomerular (PCs) and superficial axon cells (sSC), these are interneurons that establish bidirectional contacts with other regions in the OB [41]. PCs inhibit mitral cells (MCs), and this last ones communicate back to glomeruli through excitatory connections, increasing the firing of the surrounding neurons [41]. Studies have demonstrated that the connectivity of the OB neural circuits as well as olfactory performance are disturbed in mice overexpressing human APPsw, even before the onset of plaque accumulation [42].

Furthermore, in ex vivo experiments, acute incubation of the OB with oligomeric AβO increases in the spontaneous firing rate of the mitral cells [9], which in turn send excitatory inputs back to the neurons in the glomerular layer [41]. Supporting our hypothesis that the acute effects in neuronal excitability of the OB circuits might be involved in the olfactory impairments observed in the present study, Alvarado-Martínez and others [36, 43] have demonstrated alterations in the synchronized activity of the OB after AβO exposure, specifically, a decrease in the activation of the granular cell layer, which in turn inhibit the neurons in the mitral cell layer, leading to the hyperexcitability of the MC’s and the glomerular layer, ultimately leading to olfactory impairments after AβO i.c.v. and intra-bulbar administrations.

In the present study, we report a significant decrease in the amount of NeuN expressing cells in the glomerular layer of the olfactory bulb after Aβ administration, which probably correspond to periglomerular cells and the superficial axon cells [44]. Hence, we hypothesize that this decrease in NeuN might be related to the reported excitotoxic effect of Aβ [45, 46], by inducing changes in the excitatory activity or the lateral inhibitory processes that take place in the GL. Importantly, this layer is one of the first regions affected in the Tg2576 AD genetic model [8], suggesting an increased susceptibility of the GL to the effects of AβO’s.

In addition, other authors have demonstrated that i.c.v. administration of AβOs in mice decreases the number of neurons and the expression of neurotransmitters in the OB, and also increases ROS production resulting in poor olfactory performance [47]. In addition, the i.c.v. delivery of Aβ induces neuronal death mediated by caspase 3 in the rat cortex [48]. Since we did not evaluate cell death or apoptotic markers, the effects observed in NeuN could also be related to a decrease in its expression and not to cell death; future studies should address this possibility.

Although NeuN has been previously used to identify the subpopulations of neurons in the OB [49–52], not all the periglomerular cells express this marker [53]. Therefore, we believe that additional markers like doublecortin, GAD65-67, neurocalcin, tyrosine hydroxylase, among others, should be evaluated in future studies to have a more comprehensive perspective regarding the causes underlying the olfactory impairments observed after the i.c.v. administration of Aβ_1–42_.

Studies regarding the APN levels in AD patients are controversial, thus the role of this hormone in the pathogenesis of this neurodegenerative disease is not understood [30]. In the present in vivo study, we have shown that APN in the brain prevents the olfactory impairments induced by the administration of i.c.v. Aβ_1–42_. Similar results have been previously reported in a different AD rodent model induced by the i.c.v. administration of streptozotocin (STZ), where i.c.v. APN administration also improved memory [54] suggesting that central APN attenuates STZ induced cognitive impairments.

Furthermore, APN receptors are expressed in the OB [55] and this hormone can enhance the neuronal activation of the neurons in the OB in response to odors [56], which suggests that APN may have a direct effect in olfactory-related regions in the central nervous system.

The protective effects of APN could be due to its neurotrophic and anti-inflammatory role, therefore acting as a neuroprotective agent and thus preventing Aβ_1–42_ impairments.

APN is known to promote neurogenesis during embryonic development and adulthood due to its neurotrophic properties [57, 58]. This is in accordance with our data that shows a significant increase in NeuN immunoreactivity in the glomerular layer of the APN-treated rats. In addition, previous reports have demonstrated that APN deficiency reduces the generation of adult granule neurogenesis by suppressing neural progenitor cell proliferation and differentiation [24].

In contrast, i.c.v. APN administration increases neural progenitor cell proliferation in the DG of adult C57BL/6J mice [57]. Furthermore, restoring hippocampal APN levels through exercise in STZ-treated mice (which also present AD like cognitive impairments) restores the number of Ki67 and doublecortin (DCX) positive cells and the ratio of co-labeling of DCX as well as bromodeoxyuridine (BrdU) in the DG [59].

Finally, AdipoR1 activation protects from cell damage and synaptic dysfunction in the brain during hyperglycemia by regulating the survival, proliferation and differentiation of neural stem cells [60]. This proliferative effect of APN is in accordance with our data that shows a significant increase in NeuN immunoreactivity in the glomerular layer of the APN-treated rats and the prevention of the loss of NeuN immunoreactivity in the Aβ-APN group, suggesting that the effects of APN in the OB could be related to this property.

There are no studies determining if APN enhances the proliferation of the neuronal precursors in the sub-periventricular zone (SVZ), which is the region responsible for the production of neuroblast precursors that migrating to the OB to differentiate into interneurons [61, 62]. Further studies should address whether APN enhances the proliferation of neuroblasts in the SVZ and if these precursors indeed differentiate into neurons in the GL.

One of the limitations of the present study is that we did not evaluate any of the genes/proteins that could indicate if processes such as migration or differentiation were t responsible for the increase in NeuN in both APN and APN–Aβ treated rats. Further studies should address the mechanisms involved in the effects of i.c.v. APN in the neurons of the OB.

Olfactory detection tasks are attributed to a circuit in the OB, while odor discrimination, odor formation and storage of olfactory memory also involve an important olfactory-hippocampal pathway [63, 64]. We also evaluated NeuN and Iba-1 expression in the HIPP and observed an increase in the number of Iba-1 expressing cells in CA1, CA3 and DG of animals treated with Aβ_1–42_ that was prevented by the administration of APN prior to Aβ_1–42_. The number of cells expressing Iba-1 in the OB did not present changes.

This is one of the few in vivo reports that demonstrate that APN administration in the brain changes Iba-1 expression, which coincides with in vitro data showing that this hormone exerts an important anti-inflammatory effect by preventing microglial neurotoxic activation to phenotype M1; thus decreasing the expression of pro-inflammatory cytokines like Tumor necrosis factor-α (TNF-α) and interleukin1-β (IL-1β) in response to the acute exposure to AβOs [26, 65, 66]. This suggests that APN in the brain polarizes microglia to the anti-inflammatory M2 phenotype.

In the present paper, we have shown that APN administration increases the amount of ARG-1 in both the OB and the HIPP. This marker is an M2 microglial marker expressed when these cells are activated by anti-inflammatory signals like interleukin-10 (IL-10), transforming growth factor-β (TGF-β), IL-13 amongst others [67].

Microglial M2 activation is characterized by the secretion of anti-inflammatory cytokines and increased phagocytosis for debris removal, thus promoting reparation and neuroprotection [55]. APN inhibits the activation of the nuclear factor-κB (NF-κB) pathway and the cluster of differentiation 68 (CD68), therefore inhibiting the synthesis of IL-1β, IL-6, and TNF-α in vivo and in vitro, and preventing the cytotoxic actions of microglia [26, 27, 66]. In this sense, APN has clear pleiotropic, including both anti- and pro-inflammatory outcomes.

The effects of APN on the immune response appear to be highly dependent on the isoform and its target tissue [68]. While HMW and MMW isoforms strongly induce chemokine and pro-inflammatory cytokine secretion in rheumatoid arthritis synovial derived fibroblasts, LMW promotes minimal chemokine and cytokine expression [69].

Regarding immune cells, APN administration has shown opposite effects. Studies performed by Wilk et al., show that APN significantly decreases antigen-specific T cell proliferation and cytokine production [70]. In addition APN-treated dendritic cells have low production of IL-12p40, CD80, CD86, and the major histocompatibility complex class II (MHCII), suggesting a immunomodulatory effect [71]. On the contrary, the administration of APN to polyclonally activated CD4+ T cells enhances proinflammatory cytokine production, thus promoting Th differentiation, which supports its pro-inflammatory role [72].

Furthermore, HMW APN is thought to be responsible for vascular protection through the suppression of TNF-α endothelial secretion [73, 74]. In contrast, globular APN mainly promotes inflammation in cardiomyocytes during acute cardiac disease. Interestingly, this isoform has been reported to inhibit microglial M1 activation via AdipoR1 [75]. In the current study we observed a significant increase in ARG-1 expression, a M2 microglial marker, accompanied with the decrease in the amount of Iba-1 positive cells, suggesting that the effect of our current experimental approach is mainly anti-inflammatory.

Other reports using the same APN brand that we employed show both beneficial and detrimental effects depending on the pathology and the tissue [76–78], suggesting that this recombinant human adiponectin may contain several oligomeric isoforms, and that in the CNS it promotes an anti-inflammatory outcome [75].

Finally, AdipoR1 and AdipoR2 have been reported in both the HIPP an OB [55, 79], thus, we hypothesize that the effects of APN could be mediated through these receptors, since previous studies in our group have demonstrated that i.c.v. APN administration in healthy rats increases the expression of insulin receptors (InsR) [55]. Given that AD patients tend to present hyperinsulinemia and decreased insulin sensitivity [40, 41], we believe that the beneficial effects of APN in both the OB and the HIPP are mediated by the sensitization of InsR. Future studies should aim to test this hypothesis.

## Conclusion

In conclusion, we have demonstrated that the olfactory damage caused by the i.c.v. administration of Aβ can be prevented by increasing APN levels in the central nervous system, suggesting that this hormone might prevent the damage associated with Aβ acute administration in our Aβ_1–42_ model. Moreover, we propose further studies aimed to investigate the possible use of APN-based therapies in order to prevent or even ameliorate the progression of AD and other neurodegenerative diseases.

## Methods

### Animals

This study was conducted in accordance with the guidelines and requirements of the World Medical Association Declaration of Helsinki (1964), approved by the local ethics committee (protocol IN 2015716 Facultad de Medicina, UNAM), and regulated by the Mexican Official Norm NOM-062-ZOO-1999 to minimize animal suffering.

A total of 48 male Wistar rats, 40 adults (248.42 g ± 12.6 g weight) and 8 juveniles (112 ± 6.48 g weight) from the Faculty of Medicine vivarium, UNAM, were used for the experiments. Animals were maintained under a 12 h light–dark regime (lights were on from 7 a.m. to 7 p.m.) with water and standard laboratory chow ad libitum (PMI Nutrition International Inc., Greenwood, MO).

### Stereotaxic surgery

All subjects were anesthetized with a ketamine/xylazine mixture (15 mg/kg + 1 mg/kg, ip) for i.c.v. stereotaxic surgery, which was performed in a standard rodent stereotaxic frame (David Kopf, USA).

The animals were divided into 4 groups of 8 each: the first group (VEH) was injected with 1 μl of isotonic saline solution (SS) followed by 5 μl of the same solution 30 min later; a second group (APN) was injected with 1 μl of adiponectin [150 ng/μl Adiponectin/Acrp (unconjugated) 1065-AP-050, Novus Biologicals] [65] followed by 5 μl of SS 30 min later; the third group (Aβ) was first injected with 1 μl SS and 30 min later with 5 μl of Aβ_1–42_ (1 μg/μl, Amyloid β protein fragment 1–42, Sigma, A9810, dissolved in SS pre-incubated at 37 °C for 72 h in a shaking-water bath to induce aggregation); the last group (APN–Aβ) was administered with 1 μl of APN and 5 μl of Aβ_1–42._ The stereotaxic coordinates employed for i.c.v. injection were measured as 0.92 mm posterior to bregma; 1.5 mm lateral to sagittal suture; 3.9 mm beneath the brain surface [80]. After surgery, animals were housed individually in an acrylic box cage (50 × 50 × 42 cm), and behavioral tests were performed 24, 48 and 72 h after the surgery as previously reported [54]. Animals that did not show alertness and proper motility were discarded from the study.

## Experimental design

### Olfactory tests

#### Social recognition test

The social recognition test was performed as described previously [81–83]. Briefly, 2 days prior to the experiments and just before the test, adult rats were daily habituated to the testing cage (50 × 50 × 42 cm) for 4 min. Animals were tested 24 h after the second i.c.v. injection. Only the animals interested in exploring the juveniles were included for posterior analysis (VEH n = 7, APN n = 6, Aβ_1–42_ n = 8, APN–Aβ_1–42_, n = 6).

Each testing session comprised a sequence of three trials of 4 min each. The time between trials was 10 min, and each trial was recorded for later analysis. During the testing day, the experimental groups were subjected to the three trials: the first one was a habituation period to the testing cage for the adult experimental rats, where we placed two black round metal empty cages (diameter 10 cm × tall 16 cm) which would serve to enclose the juvenile rats in the following trials. These cages allowed the adult experimental rat to watch and smell, but not touch the juvenile male inside. During the second trial, rats were introduced to the testing cage together with the first unfamiliar juvenile (Training) enclosed in one of the two black metal cages (social memory acquisition); the third trial was a re-exposure to the first juvenile (Familiar) together with a second unfamiliar juvenile (Novel), both simultaneously presented inside the two black metal cages; this third trial was performed 60 min after the social memory acquisition. Following each test, the cage was thoroughly cleaned with 70% ethanol. Video recording of investigatory behavior was used to assess the time spent by adult rats investigating the stimulus animal in the social recognition test. We quantified the time that the animals spent investigating the unfamiliar and the familiar juveniles and the time the animals explored the black metal cages during the habituation period.

#### Habituation–dishabituation test

Habituation–dishabituation behavior is also used for the assessment of olfactory memory by observing the ‘rewarded olfactory discrimination’ ability of subjects. Animals were tested 24 h after the i.c.v. injection. The habituation/dishabituation test was performed using histological cartridges holding cotton swab scented with different extracts, corresponding to complex odors that have been previously reported for this test [81, 83, 84]. We only included the animals that were interested in exploring the water cartridges at the beginning of the test (VEH n = 8, APN n = 5, Aβ n = 7, APN–Aβ n = 5).

Habituation was done in a different testing room from the one where we prepared the scented cottons. We placed a small cotton ball into a clean cartridge with 50 μl of coconut extract (DEIMAN) and placed it inside the testing cage for 30 s 3 consecutive times with a 10 min intervals between each placement. Later, we performed the same procedure for a second scent, banana extract (DEIMAN). The manipulation of the cartridges was done with different gloves for each scent.

Every trial was recorded and blindly evaluated, we quantified the time rats spent sniffing the cartridges in each trial, determining sniffing as the moment the animals pointed the nose towards the cartridges and a clear movement of the whiskers was observed. We also considered picking up the cartridges with the paws as sniffing behavior.

#### Block test

This second social recognition test was performed 48 h after the surgeries, the subjects were exposed to blocks from their own cage and from a non-self cage. Exploration time for each block was quantified, following previously reported procedures for mice [84–86] with minor modifications to make the test suitable for rats. Only the animals interested in exploring the cubes were included (VEH n = 7, APN n = 6, Aβ n = 6, APN–Aβ n = 8).

Briefly, 4 plastic blocks (labeled A–D, 1-inch × 1-inch × 1-inch) were placed inside the clean home cages of individually housed rats 24 h before the test. 1 h prior to testing, the blocks were removed and placed inside Ziploc bags on top of the housing cages for one hour. In the first six trials, we placed each animals’ own A, B and C blocks, then, we removed the blocks from the cage and placed them back into the bag; this was done 6 times with intervals of 5 min between each trial. On the 7th trial, we followed the same procedure, except that instead of placing block C from the rat’s own cage, we replaced it with block D from another rat’s cage. We determined the time the animals explored the alien block, as compared to that of blocks A–B. The manipulation of the blocks was done with different gloves for each rat.

### Animal euthanasia

72 h after the i.c.v. injections, rats were anaesthetized with an overdose of sodium pentobarbital (Sedalphorte, 65 mg/ml) and perfused with 50 ml of 0.9% saline followed by 250 ml of fixative 4% paraformaldehyde in phosphate buffer saline (PBS, 0.1 M, pH 7.2). The time between anesthesia and fixation was 5 min. Brains were removed and post-fixed for 24 h in paraformaldehyde 4%. After post fixation, brains were cryoprotected in 30% sucrose for 3–4 days for further immunohistochemistry analysis.

### Histological assessments

#### Immunohistochemistry

Brains were frozen and cut at − 18 °C. Serial sections (40 µm) were coronally cut with a cryostat (LEICA, CM1850) and collected in PBS 0.1 M with 0.1% sodium azide.

In order to stain mature neurons, a primary antibody against NeuN (monoclonal mouse anti-NeuN, Abcam Cat #ab104224; 1:500) and a biotinylated secondary antibody (anti-mouse, made in horse Vector; 1:500) were used. The ABC and peroxidase substrate kits (Vector) were used to visualize bound antibody and the sections were further stained with diaminobenzidine.

To stain microglial cells, a primary antibody against Iba-1 (monoclonal goat anti-Iba-1, Abcam Cat #ab5076; 1:1000) and a biotinylated secondary antibody (anti-goat, made in donkey Jackson; 1:500) were used. The ABC and peroxidase substrate kit (Elite, Vector) was used to visualize bound antibody and the sections were further stained with diaminobenzidine.

The neurons in the granular cell layer (GCL) and the glomerular layer (GL) in the OB, and CA1, CA3, hilus and DG in the HIPP were identified and evaluated using a NIKON microscope (eclipse 50i). The images were digitalized with a DS-U2 S Camera at 5 and 20× (Plan-Apochromat®, 1.25 NA/160).

The latter quantifications were performed on gray-scale images with a 20× objective lens and computer-assisted imaging analysis system (Image J) software (National Institutes of Health, USA).

#### Immunohistological analysis

We evaluated the number of cells per area in 3 different rectangular selections inside the granular and glomerular layers of the OB and the CA1, 3, hilus and DG in the HIPP, respectively. The placement of the 3 rectangles inside each region of interest was randomly assigned by creating 4 different personalized Macros in ImageJ/FIJI (see Fig. 2 for the OB regions and S2 for the HIPP regions which include examples of the rectangle placements). These Macros allowed us to automatize the quantification areas, instead of manually placing them in order to ensure an unbiased quantification of the number of cells in each one of the regions. We quantified 4 sections of each region including left and right of six rats per group and the average per rat was reported, this evaluation was performed by 2 evaluators blind to the treatments.

### Western blot

Protein extraction of 4 rats per group was performed using NP-40 lysis buffer containing protease inhibitors (Complete, Roche). 30 μg of protein per condition was run on 10% SDS-PAGE gels and then transferred to nitrocellulose membranes (BioRad) which were subsequently blocked for 1 h with a solution of 3% BSA in TBS/0.05% Tween or 5% skim milk in TBS/0.05% Tween (for β-Amyloid). Membranes were incubated overnight with primary antibodies against Arginase 1 (1:8000, PA5-18684 Invitrogen), β-Amyloid (1:1000, sc-53822 Santa Cruz Biotechnology) and β-Actin (1:5000, GTX629630 GeneTex). Subsequent rinsing was performed with TBS/0.05% Tween and then membranes were incubated for 1 h with anti-goat or anti-mouse secondary antibodies diluted 1:10,000 (Santa Cruz Biotechnology), followed by a second rinsing with TBS/0.05% Tween. The presence of proteins was revealed by chemiluminescence (WBLUF0500, Millipore). The images were captured using MiniBIS Pro imaging system (DNR Systems Imaging-Bio Ltd.). Densitometry analysis was performed using Image J 1.52a software.

### Statistical analysis

Social recognition test was evaluated with a Students T test, habituation–dishabituation, the block tests and WB were evaluated by performing a One-way ANOVA with a post-hoc Tukey test. NeuN and Iba-1 data were evaluated with a One-way ANOVA with a post-hoc Tukey test. Comparisons between Intact and vehicle treated animals were done using a series of unpaired Student’s T tests. Results were considered to be statistically significant if p ≤ 0.05*.* Graphs and statistical analyses were elaborated using the GraphPad Prism version 8 for macOS, GraphPad Software, La Jolla, California, USA.

## Supplementary Information

**Additional file 1: Figure S1.** Olfactory tests in intact and vehicle-treated animals. (A) Social recognition test for intact and vehicle (VEH) treated rats, unpaired Student’s T test for Novel-1 intact vs. VEH: T = 1.43, df = 15, p = 0.2962, Familiar intact vs. VEH: T = 1.702, df = 15, p = 0.6413 and Novel-2 intact vs. VEH: T = 1.396, df = 15, p = 0.9322. (B) Block test, unpaired Student’s T test for block A intact vs. VEH: T = 1.405, df = 15, p = 0.18, block B vs. VEH: T = 0.1823, df = 15, p = 0.8578 and block C intact vs. VEH: T = 0.7088, df = 15, p = 0.4893. (C) Habituation–dishabituation test (Two-way ANOVA interaction: F_[8, 54]_ = 0.58, p = 0.7849, Sidak’s multiple comparisons post hoc test did not show significant differences). Data are presented as SEM.

**Additional file 2: Figure S2.** NeuN and IBA-1 representative micrographs for CA1, CA3, hilus and the dentated gyrus of the hippocampus. CA1 (scale bar for NEUN 100 µm IBA-1 150 µm), CA3 (scale bar for 50 µm), hilus (scale bar for NeuN 100 µm and for IBA-1 80 µm), DG (scale bar for NEUN 50 µm and for IBA-1 100 µm).

**Additional file 3: Figure S3.** Full-length blots of Aβ oligomer detection in the olfactory bulb and hippocampus. (A) Aβ oligomer blots of the experimental groups Veh (vehicle), Aβ_1–42_, APN (adiponectin) and APN + Aβ_1–42_. (B) Aβ oligomer blots of the experimental groups with increased exposition. (C and D) Corresponding β-Actin blots of the experimental groups. Densitometryc analysis was performed using A and D images for olfactory bulb and B and C images for hippocampus samples.

**Additional file 4: Figure S4.** Full-lenght blots of ARG1 and GFAP detection in the olfactory bulb and hippocampus. (A) ARG1 and GFAP blots of the experimental groups Veh (vehicle), Aβ_1–42_, APN (adiponectin) and APN + Aβ_1–42_. ARG1 (37KD) and GFAP (50 KD) were run in the same blots. (B) Corrsponding β-Actin blots of the experimental groups. HP: hippocampus, BO: olfactory bulb.

## Data Availability

All data generated or analyzed during this study are included in this published article.

## Abbreviations

AD: Alzheimer’s disease
AdipoR: Adiponectin receptor
Aβ: Amyloid beta
AβO: Aβ oligomers
Aβ_1–42_: Amyloid-beta_1–42_
AβPP/PS1: Familial mouse model of AD
APN: Adiponectin
APN–Aβ_1-42_: Adiponectin–amyloid-beta_1–42_
APN-KO: APN knockout
APPsw: APP transgenic mouse, also known as Tg2576
ARG-1: Arginase-1
BV2: Microglial cell line
BrdU: Bromodeoxyuridine
CD68: Cluster of differentiation 68
CSF: Cerebrospinal fluid
DG: Dentate gyrus
DCX: Doublecortin
Fig.: Figure
GCL: Granule cell layer
HIPP: Hippocampus
HT-22: Hippocampal neuronal cell line
i.c.v.: Intracerebroventricular
Iba-1: Ionized calcium-binding adapter molecule 1
IL-1β: Interleukin1-β
IL-10: Interleukin-10
InsR: Insulin receptor
IRS-1: Receptor substrate-1
LMW: Low molecular weight
M1: Microglial neurotoxic activation
M2: Microglial anti-inflammatory activation
MCs: Mitral cells
MHCII: Major histocompatibility complex class II
MMW: Middle molecular weight
NeuN: Neuronal nuclei
NF-κB: Nuclear factor-κB
OB: Olfactory bulb
PC: Periglomerular cells
p-TAU: Phosphorylated microtubule-associated protein tau
sSA: Superficial axon cells
SS: Saline solution
STZ: Streptozotocin
SVZ: Sub-periventricular zone
TGF-β: Transforming growth factor-β
TNF-α: Tumor necrosis factor-α
VEH: Vehicle
