# Correction to: Protective effects of intracerebroventricular adiponectin against olfactory impairments in an amyloid β_1–42_ rat model

**DOI:** 10.1186/s12868-021-00630-7

**Published:** 2021-04-20

**Authors:** Mara A. Guzmán-Ruiz, Amor Herrera-González, Adriana Jiménez, Alan Candelas-Juárez, Crystal Quiroga-Lozano, Claudia Castillo-Díaz, Erika Orta-Salazar, Diana Organista-Juárez, Sofía Díaz-Cintra, Rosalinda Guevara-Guzmán

**Affiliations:** 1 Departamento de Fisiología, Facultad de Medicina, Universidad Nacional Autónoma de México (UNAM), Mexico City, Mexico; 2 Departamento de Neurobiología del desarrollo y neurofsiologíaInstituto de Neurobiología, Universidad Nacional Autónoma de México (UNAM), Querétaro, Mexico

## Correction to: BMC Neurosci 22:14 (2021) https://doi.org/10.1186/s12868-021-00620-9

Following publication of the original article [1], it was reported that a duplicate image of Fig. 3 was published for Fig. 5. The correct Fig. 5 is included in this Correction article and the original article has been updated.

**Fig. 5:**
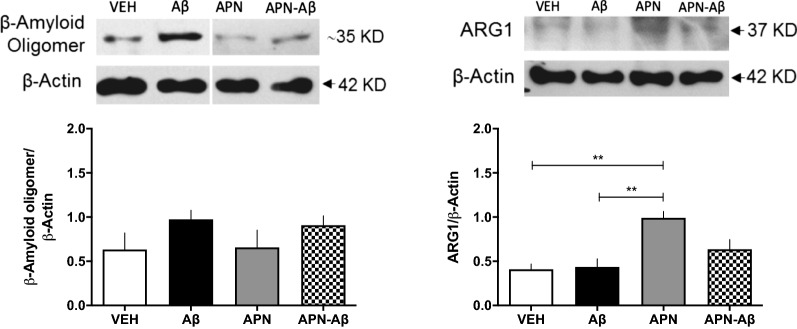
Protein expression in the hippocampus. **a** Representative western blot of β-Amyloid oligomer and β-Actin; **b** β-Amyloid/β-Actin ratio; **c** representative western blot of Arginase1 (ARG1) and β-Actin; **d** ARG1/β-Actin ratio for the vehicles (VEH), adiponectin (APN), Amyloid-β (Aβ) and APN–Aβ injected rats. Data are presented as SEM and evaluated using a One-way ANOVA with a post-hoc Tukey test
