# Correction: Protective effects of intracerebroventricular adiponectin against olfactory impairments in an amyloid β_1–42_ rat model

**DOI:** 10.1186/s12868-023-00815-2

**Published:** 2023-08-28

**Authors:** Mara A. Guzmán‑Ruiz, Amor Herrera‑González, Adriana Jiménez, Alan Candelas‑Juárez, Crystal Quiroga‑Lozano, Claudia Castillo‑Díaz, Erika Orta‑Salazar, Diana Organista‑Juárez, Sofía Díaz‑Cintra, Rosalinda Guevara‑Guzmán

**Affiliations:** 1 Departamento de Fisiología, Facultad de Medicina, Universidad Nacional Autónoma de México (UNAM), Mexico City, Mexico; 2 Departamento de Neurobiología del desarrollo y neurofisiología, Instituto de Neurobiología, Universidad Nacional Autónoma de México (UNAM), Querétaro, Mexico

**Correction: BMC Neurosci (2021) 22:14 ** 10.1186/s12868-021-00620-9

Following publication of the original article [1], the authors identified errors in Fig. 2b and e and Additional file 1: Fig. S2. In Fig. 2b we have now changed the APN-βA representative figure for IBA1 micrographs. In the original figure the same APN-βA section for Fig. 2b, e since it was the best preserved section. In Fig. 2b the focus was directed to the granular layer, while in Fig. 2e to the glomerular layer, the image was flipped so all the glomeruli could appear on top of the picture.

In the second panel of the IBA1 micrographs in Fig. 2e, the animal from the APN-βA was repeated instead of employing the corresponding representative section for the APN group, now the group has been corrected.

This mistake was done while elaborating the figures but do not affect the original published results.

The correct Fig. 2 is given in this erratum.

**Fig. 2:**
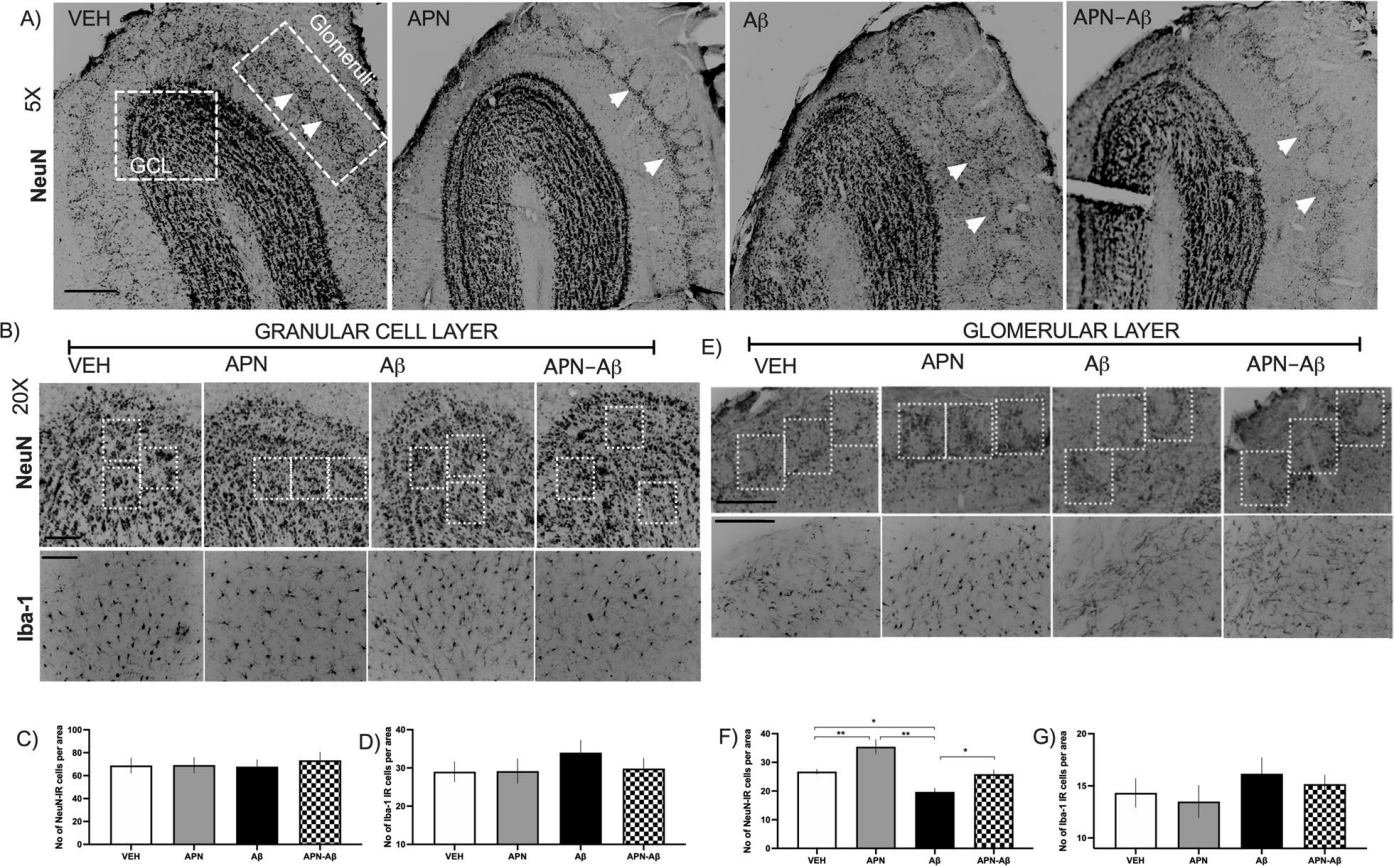
Immunohistological determination of NeuN and Iba-1 expression in the olfactory bulb. **a** Representative micrographies of the olfactory bulb showing the evaluated areas for NeuN and Iba‐1, scale bar 250 μm; the arrows denote the region where glomeruli are disarranged. **b** Representative micrographies of NeuN (top) and Iba‐1 (bottom) of the granular cell layer (GCL) with examples of the random squares (ROIs) quantified, scale bars 50 μm. **c** Number of NeuN expressing cells in the GCL for Vehicles (VEH), adiponectin (APN), Amyloid‐β (Aβ) and APN-Aβ injected rats (F_[3, 20]_ = 0.1, p < 0.94). **d** Number of Iba‐1 positive cells per area of the GCL of the same groups (F_[3, 20]_ = 0.6336, p < 0.602). **e** Representative micrographies of the glomeruli with examples of the random squares (ROIs) quantified, scale bar 50 μm. **f** Number of NeuN expressing cells in the glomeruli per area of the same groups (F_[3, 68]_ = 15.93, p < 0.0001). **g** Number of Iba‐1 positive cells per area (F_[3, 20]_ = 0.7029, p < 0.5614. Data are presented as SEM and evaluated using a One‐way ANOVA with a post‐hoc Tukey test

In Additional file 1: Fig. S2 the micrograph employed for representing the APN and APN-βA groups were different pictures but from the same animal. This mistake has been corrected.

It is important to highlight that the original images have been rotated so the sections can be appreciated in the same orientation. This was necessary since we perform free floating immunohistochemistry, therefore, when the sections are placed in the gelatinized slides some of them tend to be placed in different orientations. In addition, the microscope that we use is unable to rotate the stage.

However, since there were no significant differences in the number of NeuN+ cells, this mistake does not affect the original published results.

The correct Additional file 1: Fig. S2 is given in this erratum.

Figure 2 and Additional file 1: Fig. S2 has been updated in this Correction article.

### Supplementary Information

**Additional file 1: Figure S2.** NeuN and IBA-1 representative micrographs for CA1, CA3, hilus and the dentated gyrus of the hippocampus. CA1 (scale bar for NEUN 100 μm IBA-1 150 μm), CA3 (scale bar for 50 μm), hilus (scale bar for NeuN 100 μm and for IBA-1 80 μm), DG (scale bar for NEUN 50 μm and for IBA-1 100 μm). 
