# Supplementary figures and images for: Protective effects of intracerebroventricular adiponectin against olfactory impairments in an amyloid β1–42 rat model
Source: BMC Neurosci. 2021 Mar 2;22:14. doi: 10.1186/s12868-021-00620-9 (PMC7927416; doi:10.1186/s12868-021-00620-9)

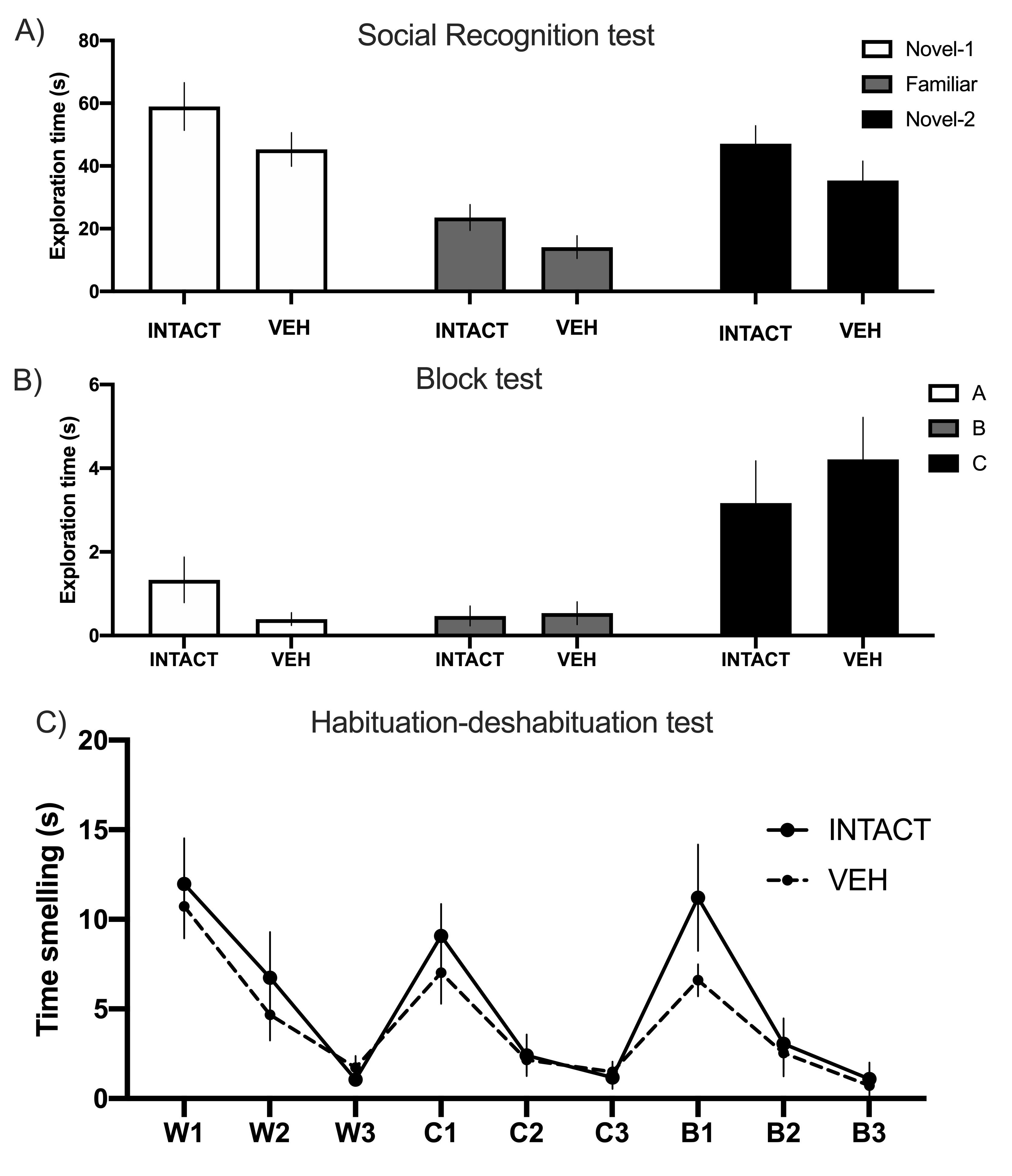

Supplement: Supplementary file 1 — Additional file 1: Figure S1. Olfactory tests in intact and vehicle-treated animals. (A) Social recognition test for intact and vehicle (VEH) treated rats, unpaired Student’s T test for Novel-1 intact vs. VEH: T = 1.43, df = 15, p = 0.2962, Familiar intact vs. VEH: T = 1.702, df = 15, p = 0.6413 and Novel-2 intact vs. VEH: T = 1.396, df = 15, p = 0.9322. (B) Block test, unpaired Student’s T test for block A intact vs. VEH: T = 1.405, df = 15, p = 0.18, block B vs. VEH: T = 0.1823, df = 15, p = 0.8578 and block C intact vs. VEH: T = 0.7088, df = 15, p = 0.4893. (C) Habituation–dishabituation test (Two-way ANOVA interaction: F[8, 54] = 0.58, p = 0.7849, Sidak’s multiple comparisons post hoc test did not show significant differences). Data are presented as SEM. [file 12868_2021_620_MOESM1_ESM.tiff]

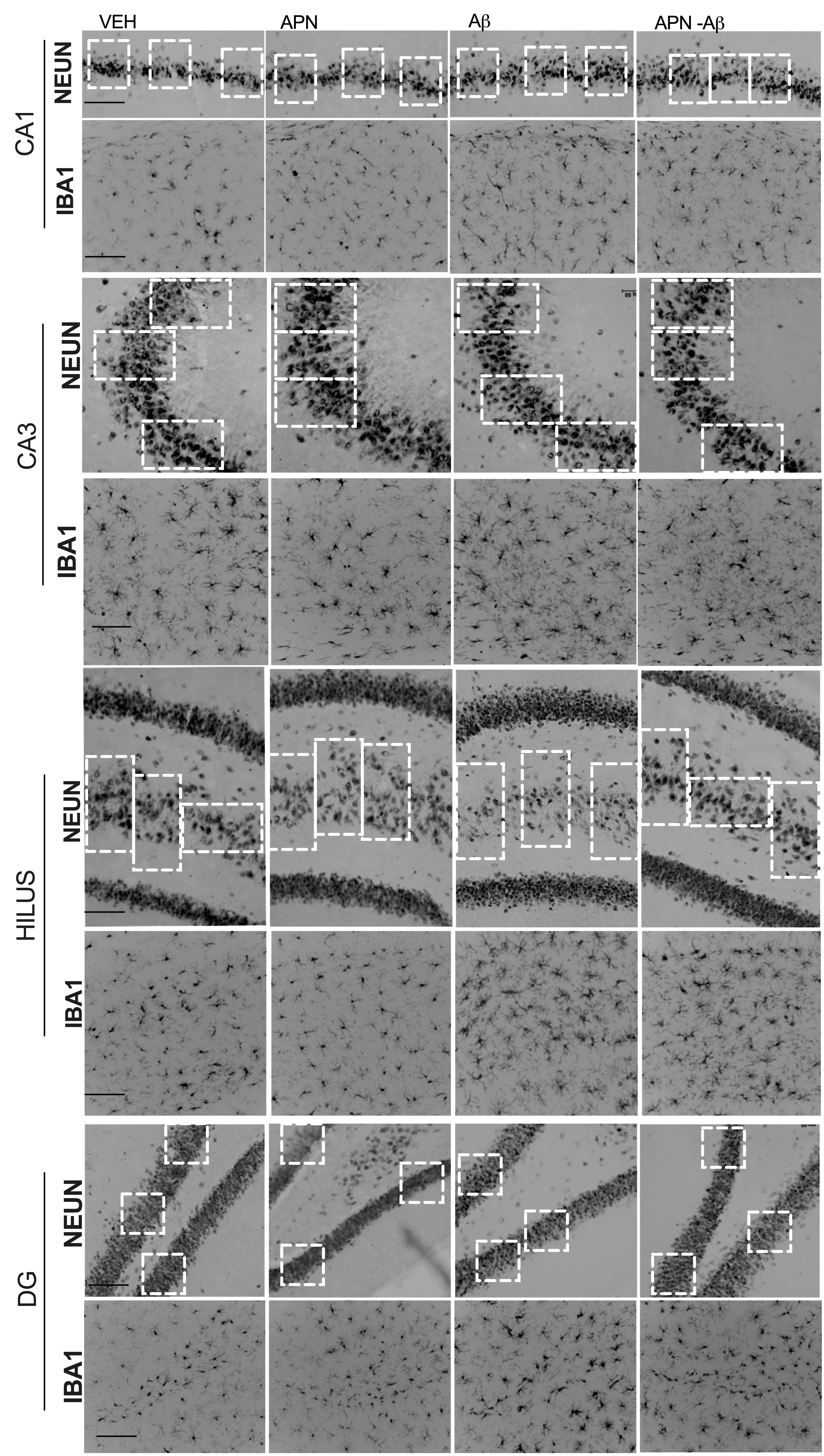

Supplement: Supplementary file 2 — Additional file 2: Figure S2. NeuN and IBA-1 representative micrographs for CA1, CA3, hilus and the dentated gyrus of the hippocampus. CA1 (scale bar for NEUN 100 µm IBA-1 150 µm), CA3 (scale bar for 50 µm), hilus (scale bar for NeuN 100 µm and for IBA-1 80 µm), DG (scale bar for NEUN 50 µm and for IBA-1 100 µm). [file 12868_2021_620_MOESM2_ESM.tiff]

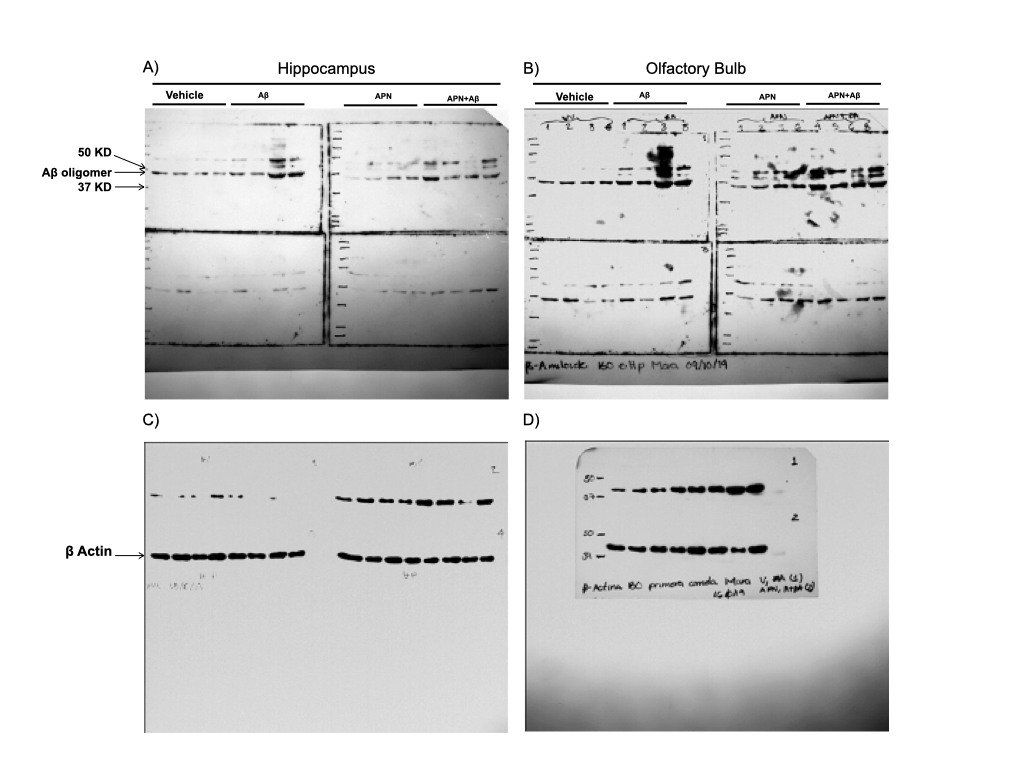

Supplement: Supplementary file 3 — Additional file 3: Figure S3. Full-length blots of Aβ oligomer detection in the olfactory bulb and hippocampus. (A) Aβ oligomer blots of the experimental groups Veh (vehicle), Aβ1–42, APN (adiponectin) and APN + Aβ1–42. (B) Aβ oligomer blots of the experimental groups with increased exposition. (C and D) Corresponding β-Actin blots of the experimental groups. Densitometryc analysis was performed using A and D images for olfactory bulb and B and C images for hippocampus samples. [file 12868_2021_620_MOESM3_ESM.tiff]

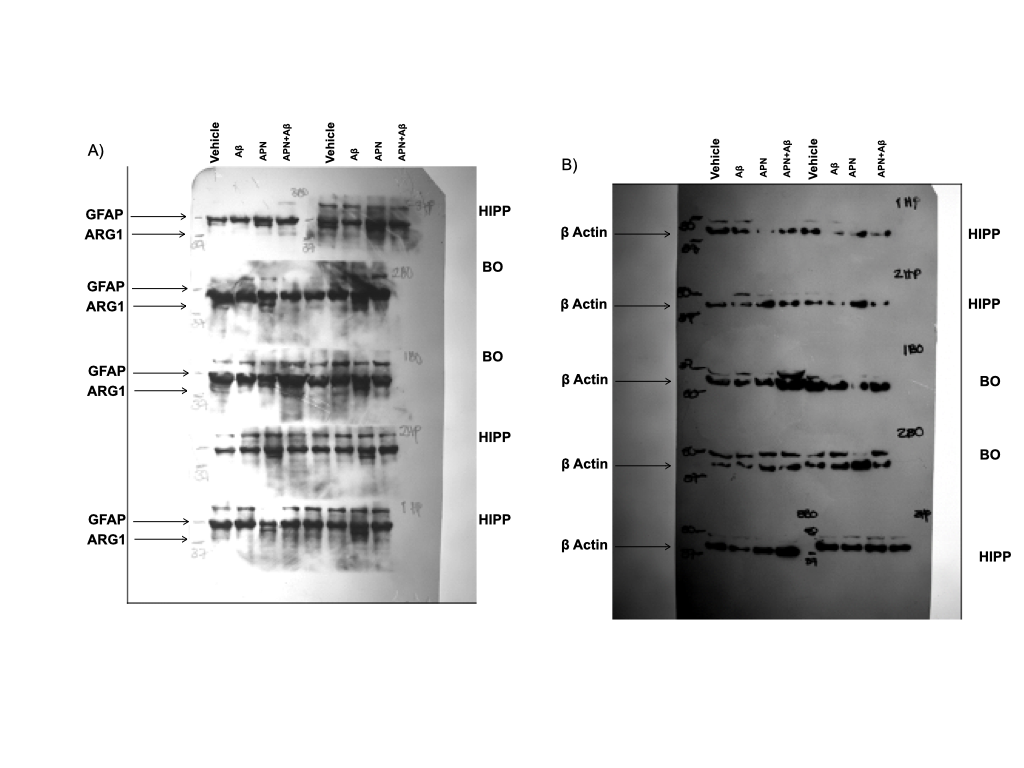

Supplement: Supplementary file 4 — Additional file 4: Figure S4. Full-lenght blots of ARG1 and GFAP detection in the olfactory bulb and hippocampus. (A) ARG1 and GFAP blots of the experimental groups Veh (vehicle), Aβ1–42, APN (adiponectin) and APN + Aβ1–42. ARG1 (37KD) and GFAP (50 KD) were run in the same blots. (B) Corrsponding β-Actin blots of the experimental groups. HP: hippocampus, BO: olfactory bulb. [file 12868_2021_620_MOESM4_ESM.tiff]
