# Supplementary figures and images for: Correction: Protective effects of intracerebroventricular adiponectin against olfactory impairments in an amyloid β1–42 rat model
Source: BMC Neurosci. 2023 Aug 28;24:47. doi: 10.1186/s12868-023-00815-2 (PMC10464160; doi:10.1186/s12868-023-00815-2)

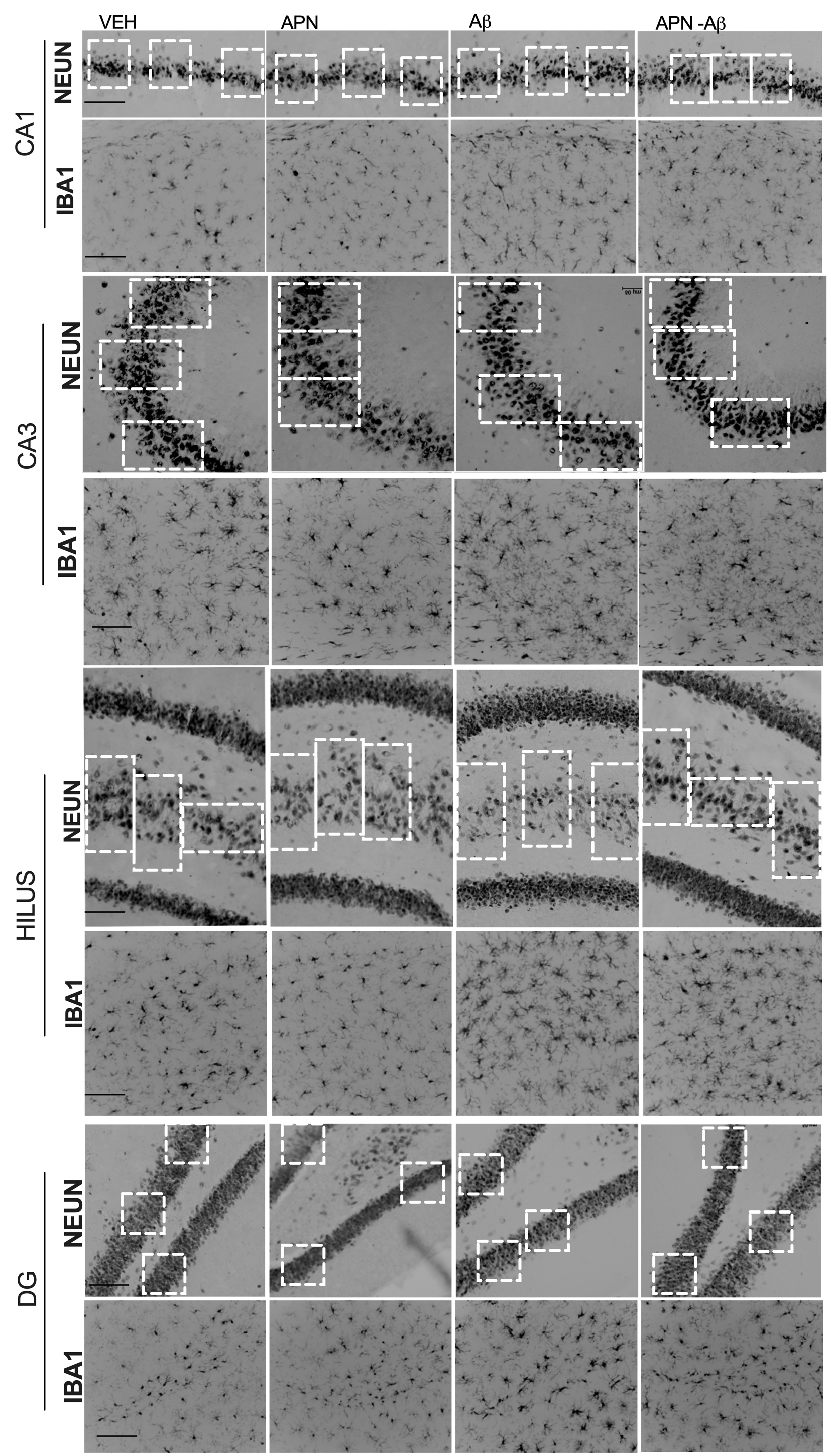

Supplement: Supplementary file 1 — Additional file 1: Figure S2. NeuN and IBA-1 representative micrographs for CA1, CA3, hilus and the dentated gyrus of the hippocampus. CA1 (scale bar for NEUN 100 μm IBA-1 150 μm), CA3 (scale bar for 50 μm), hilus (scale bar for NeuN 100 μm and for IBA-1 80 μm), DG (scale bar for NEUN 50 μm and for IBA-1 100 μm). [file 12868_2023_815_MOESM1_ESM.tiff]
